# High-affinity chicken scFv antibodies recognize recombinant VP1-CDEF' protein and native virions of feline calicivirus genotype II strain LZ2023

**DOI:** 10.3389/fmicb.2026.1896418

**Published:** 2026-09-23

**Authors:** Yanquan Wei, Yihan Liu, Zhen Han, Huitian Gou, Kaiqi Lian, Dongdong Sun, Yanhong Ji, Hongmei Wang

**Affiliations:** 1 College of Veterinary Medicine, Gansu Agricultural University, Lanzhou, China; 2 Sichuan Purity Pharmaceutical Co., Ltd., Chengdu, China; 3 School of Biological and Food Engineering, Anyang Institute of Technology, Anyang, China; 4 State Key Laboratory of Medicinal Chemical Biology and College of Life Sciences, Nankai University, Tianjin, China; 5 State Key Laboratory for Animal Disease Control and Prevention, Lanzhou Veterinary Research Institute, Chinese Academy of Agricultural Sciences, Lanzhou, China

**Keywords:** feline calicivirus, phage display, single-chain variable fragment, biolayer interferometry, engineered antibody

## Abstract

Feline calicivirus (FCV) is a significant pathogen responsible for upper respiratory tract disease and oral ulceration in felids. Genetic and antigenic variation in circulating FCV strains limits the utility of current diagnostic assays, and no validated genotype II-specific antibodies exist for rapid test development. In this study, we targeted the C, D, E, and partial F regions of the major capsid protein VP1 (termed VP1-CDEF'). The recombinant VP1-CDEF' protein was expressed in a prokaryotic system and used to immunize SPF chickens. A chicken-derived phage display single-chain variable fragment (scFv) library was successfully constructed, with a capacity of 5.25 × 10^7^ transformants and an insert-positive rate of approximately 80%. After three rounds of solid-phase biopanning, the phage recovery ratio increased from 3.6 × 10^−6^ to 6.4 × 10^−3^, representing an enrichment factor of 1,777.78-fold. Phage-ELISA screening of 184 randomly selected clones yielded 25 positive clones, and sequence analysis identified five scFv antibodies with unique amino acid sequences, designated 1A2, 2B2, 2C1, 2E1, and 2G5. The scFv genes were constructed into scFv-Fc fusion proteins and expressed in Expi293F cells. Western blot analysis demonstrated that all five scFv-Fc fusion proteins specifically recognized the recombinant VP1-CDEF' protein and exhibited no cross-reactivity with feline parvovirus VP2 protein. In indirect immunofluorescence assay (IFA)-based binding assays, however, only 2B2-Fc, 2E1-Fc, and 2G5-Fc were capable of binding genotype II FCV strain LZ2023 in infected F81 cells. Virion capture ELISA using purified intact FCV particles confirmed that 2B2-Fc, 2E1-Fc, and 2G5-Fc bind to surface-accessible epitopes on native virions, whereas 1A2-Fc and 2C1-Fc showed no detectable binding. Biolayer interferometry (BLI) analysis showed that 2B2-Fc and 2E1-Fc exhibited the highest binding affinities, with equilibrium dissociation constant (KD) values of 0.046 nmol/L and 0.032 nmol/L, respectively, whereas 2G5-Fc displayed moderate affinity (KD = 65.44 nmol/L). 1A2-Fc and 2C1-Fc showed relatively weak binding (KD > 450 nmol/L). This study reports the generation of chicken-derived scFv antibodies targeting the FCV VP1-CDEF' region. The high-affinity clones 2B2 and 2E1 are promising candidates for rapid diagnostic test development and warrant further evaluation. However, further validation of their diagnostic utility will require clinical sample studies and the development of colloidal gold strips based on these scFv.

## Introduction

Feline calicivirus (FCV) is a non-enveloped, single-stranded positive-sense RNA virus belonging to the genus *Vesivirus* within the family *Caliciviridae* (Wang et al., 2025). As a highly prevalent pathogen in domestic cat populations worldwide, FCV causes a spectrum of clinical manifestations ranging from upper respiratory tract disease and oral ulceration to severe systemic disease (Karakaya-Bilen et al., 2025). Current FCV prevention and control rely primarily on vaccination. However, commercial vaccines based on classical reference strains such as FCV-255 and F9 provide only limited cross-protection against the genetically diverse field strains currently in circulation (Hofmann-Lehmann et al., 2022; Yang et al., 2024). Epidemiological investigations have indicated that genetic differences and amino acid variations in B-cell epitopes of circulating FCV strains are associated with vaccination failure (Liang et al., 2022). Moreover, FCV vaccines do not achieve sterilizing immunity, and vaccinated cats may still become infected and shed field virus (Hofmann-Lehmann et al., 2022). To date, no specific antiviral therapeutics have been approved for FCV infection, and clinical management relies largely on supportive care (Wei et al., 2024). These challenges underscore the urgent need for complementary strategies, including specific antiviral approaches and rapid point-of-care diagnostics.

The FCV major capsid protein VP1 serves as the primary antigenic determinant of the virion and can be structurally divided into six regions (A through F). Regions C and E display high sequence variability and are closely linked to immune evasion, whereas the E region functions as the principal receptor-binding domain and harbors core neutralizing epitopes (Cubillos-Zapata et al., 2020). Accumulating evidence has established that the C, D, and E regions and a partial F region of VP1 (designated VP1-CDEF') collectively constitute the dominant neutralizing epitope cluster of FCV. Two distinct linear epitopes located on the outermost surface of the E region of the capsid have been precisely mapped: a conserved non-neutralizing epitope spanning amino acids 431–435 (PAGDY) and a highly variable neutralizing epitope spanning residues 445–451 (ITTANQY) that lies within the E5’ hypervariable region (E5’ HVR) of the E region (Cubillos-Zapata et al., 2020). More recently, Li et al. (2024) demonstrated that the CDE region can effectively stimulate humoral, mucosal, and cellular immune responses in felines, with CDE-based subunit vaccination significantly reducing disease incidence upon viral challenge. Furthermore, Yang et al. (2024) identified the CE region as the optimal protective antigen region for both genotype I and genotype II strains, showing that bivalent recombinant proteins based on this region induced cross-neutralizing antibodies with titers surpassing those of a commercial vaccine. These findings identify the VP1-CDEF' region as an ideal, high-priority target for FCV-specific antibody development.

Single-chain variable fragment (scFv) antibodies, composed of heavy-chain variable region (VH) and light-chain variable region (VL) joined by a flexible peptide linker, retain full antigen-binding capacity while possessing a molecular weight approximately one-sixth that of a full-length IgG (Gezehagn Kussia and Tessema, 2024). Owing to their small size, scFvs exhibit superior tissue penetration and hold broad application potential in disease diagnosis, targeted therapy, and biosensing (Ahmad et al., 2012). Among various host species for scFv library construction, the domestic chicken has emerged as a particularly attractive system. Unlike mammalian species, chickens generate their immunoglobulin repertoire through gene conversion from single functional VH and VL germline genes, enabling amplification of essentially the complete VH and VL repertoires using only a single primer pair per region (Finlay et al., 2017). Furthermore, the phylogenetic distance between birds and mammals allows chickens to mount robust high-affinity antibody responses against highly conserved mammalian proteins that may be poorly immunogenic in mice (Finlay et al., 2005). Since Yamanaka et al. (1996) first reported the construction of a chicken-derived scFv phage display library in 1996, this platform has been successfully employed to isolate high-affinity scFv antibodies against diverse targets. For instance, Lee et al. (2007) isolated chicken scFv clones specifically reacting with SARS-CoV-infected Vero cells, and Gong et al. (2024) successfully constructed a chicken immune scFv library against *Helicobacter pylori* and isolated scFv antibodies recognizing the HpaA protein across nine clinical strains. In veterinary applications, chicken-derived scFv antibodies have been generated against canine parvovirus (Ge et al., 2020), infectious bursal disease virus (Sapats et al., 2003), and foot-and-mouth disease virus (Opperman et al., 2012), demonstrating the versatility of this platform.

Despite the extensive application of chicken-derived scFv technology for various viral pathogens, the development of chicken scFv antibodies specifically targeting the FCV VP1-CDEF' region has not been reported. Most existing studies on FCV-targeted antibodies have focused on murine monoclonal antibodies (Cubillos-Zapata et al., 2020), whereas scFv-based reagents against FCV remain unexplored. In this study, we constructed a chicken-derived phage display scFv library against the FCV VP1-CDEF' protein, screened and characterized specific scFv antibodies, and evaluated their binding reactivity with the VP1-CDEF' protein and FCV virions. The well-characterized chicken-derived scFv antibodies will facilitate the development of rapid FCV detection assays and provide new tools for FCV research.

## Materials and methods

### Materials and reagents

The pComb3XSS phagemid vector, NEB 5α F´ competent cells, *E. coli* SS320 competent cells, and M13KO7 helper phage were purchased from New England Biolabs (NEB, UK). The pET-28a prokaryotic expression vector and *E. coli* Rosetta (DE3) competent cells were purchased from Novagen (Merck KGaA, Darmstadt, Germany). *E. coli* DH5α competent cells were purchased from TransGen Biotech Co., Ltd. (Beijing, China). Human embryonic kidney Expi293F cells were purchased from Procell Life Science & Technology Co., Ltd. (Wuhan, China). The cDNA first-strand synthesis kit and 2 × Phanta Max Master Mix (for high-fidelity PCR) were purchased from Vazyme Biotech Co., Ltd. (Nanjing, China). T4 DNA ligase, restriction endonucleases NheI and XhoI, and DNA gel extraction kits were purchased from Takara Biotech Co., Ltd. (Beijing, China). Ni affinity chromatography resin and isopropyl β-D-1-thiogalactopyranoside (IPTG) were purchased from Sangon Biotech Co., Ltd. (Shanghai, China). Primers for gene amplification were synthesized by Sangon Biotech Co., Ltd. (Shanghai, China); the primer sequences are listed in Table 1. DMEM medium, Expi293F medium, and fetal bovine serum (FBS) were purchased from Thermo Fisher Scientific. The chicken peripheral blood lymphocyte isolation kit was purchased from Solarbio Science & Technology Co., Ltd. (Beijing, China). HRP-conjugated mouse anti-M13 antibody and HRP-conjugated goat anti-Fc (human IgG) antibody were purchased from Sino Biological Inc. (Beijing, China). HRP-conjugated goat anti-chicken IgG H&L antibody was purchased from Bioss Biotechnology Co., Ltd. (Beijing, China). Freund’s complete adjuvant (FCA), Freund’s incomplete adjuvant (FIA), and Cy3-conjugated anti-human IgG-Fc antibody were purchased from Sigma-Aldrich (St. Louis, MO, United States). Hoechst 33342 was purchased from Beyotime Biotechnology (Shanghai, China). Three specific pathogen-free (SPF) chickens were purchased from Vital River Laboratory Animal Technology Co., Ltd. (Beijing, China). Feline kidney F81 cells, FCV LZ2023 strain, feline parvovirus (FPV) VP2 protein, and FCV-positive serum were all preserved in the Veterinary Public Health Laboratory, College of Veterinary Medicine, Gansu Agricultural University, China.

**Table 1:** Primers used for amplification of VP1-CDEF', VH and VL genes.

Primer	Primer sequence (5′-3′)	target gene
CDEF'-F	ATGGCTAGCAAGAAGGGAGCCAAACTAGGA	VP1-CDEF' gene
CDEF'-R	GTGCTCGAGGATGACCTCTTCGCCAATG	
VH-F	CTACAAATGCCTATGCATCCGCCGTGACGTTGGACGAG	VH gene
VH-R	CCACCCGACCCACCTCCGCCCGAGCCACCACCTCCGGAGGA GACGATGACTT	
VL-F	GGCTCGGGCGGAGGTGGGTCGGGTGGAGGCGGTTCAGCRC TGACTCAGCCGKCCT	VL gene
VL-R	AAACAACTTTCAACAGTGGATAGGACGGTCAGGGTTGT	
scFv-F	TCTTGCACTTGTCACGAATTCTGCCGTGACGTTGGACGAGTCCG	scFv-Fc
scFv-R	ACAAGATTTGGGCTCCGTACGTAGGACGGTCAGGGTTGTCCCG	

The underlined region corresponds to the homologous arm sequence.

### Preparation of VP1-CDEF' recombinant protein

The VP1-CDEF' gene fragment (encompassing the C, D, E and a partial F region of the major capsid protein VP1), corresponding to residues 398–540 of the VP1 protein, was amplified by PCR using cDNA reverse-transcribed from the genomic RNA of the FCV LZ2023 strain (genotype II, GenBank accession number: PZ105981.1), a field isolate maintained in our laboratory. To facilitate directional cloning, the forward and reverse primers were designed to incorporate NheI and XhoI restriction sites, respectively (Table 1). PCR was performed under the following conditions: initial denaturation at 95 °C for 5 min; 33 cycles of 95 °C for 30 s, 58 °C for 30 s, and 72 °C for 30 s; and a final extension at 72 °C for 5 min.

The purified PCR product and the pET-28a prokaryotic expression vector were separately double-digested with NheI and XhoI. Following gel purification, the digested VP1-CDEF' insert and the linearized vector were ligated using T4 DNA ligase at 16 °C overnight, yielding the recombinant plasmid pET-28a-VP1-CDEF'. The ligation mixture was then transformed into *E. coli* DH5α competent cells. Positive transformants were identified via colony PCR and confirmed by Sanger sequencing.

The verified recombinant plasmid pET-28a-VP1-CDEF' was transformed into *E. coli* Rosetta (DE3) competent cells via heat shock transformation. A single positive colony was picked and inoculated into Luria-Bertani (LB) medium supplemented with kanamycin for scale-up culture. When the optical density of the bacterial solution at 600 nm (OD_600_) reached 0.6–0.8, IPTG was added to a final concentration of 0.5 mM to induce protein expression, with incubation performed at 16 °C for 16–18 h. Bacterial pellets were harvested by centrifugation and lysed by ultrasonication. The supernatant was collected after centrifugation of the lysate, and then incubated with Ni affinity chromatography resin at 4 °C for 2 h with gentle rotation. Non-specifically bound contaminant proteins were sequentially removed using wash buffers (20 mM Tris–HCl, 300 mM NaCl, pH 8.0) containing 50 mM imidazole and 100 mM imidazole, respectively. The target VP1-CDEF' protein was subsequently eluted using the same buffer supplemented with 500 mM imidazole. The purified VP1-CDEF' protein were separated by SDS-PAGE, and then transferred onto a polyvinylidene fluoride (PVDF) membrane via wet transfer method. After blocking with 5% skim milk, the membrane was incubated with feline FCV-positive serum (diluted at 1:1000) as the primary antibody, followed by incubation with HRP-conjugated goat anti-feline IgG H&L antibody (diluted at 1:5000, Thermo Fisher Scientific) as the secondary antibody. Finally, Western blot verification was completed after chemiluminescent color development of the membrane. The FCV-positive serum used for Western blot verification of the recombinant VP1-CDEF' protein was collected from a single healthy cat that had been vaccinated with a commercial trivalent vaccine (Zoetis, PureVax®) containing inactivated FCV. Seroconversion to FCV was confirmed by a commercial colloidal gold antibody test strip (Korea).

### Animal immunization and antibody titer detection

For primary immunization, 1 mg of recombinant VP1-CDEF' protein was thoroughly emulsified with an equal volume of FCA and administered to two SPF chickens via multipoint intramuscular injection into the pectoral muscle. Four booster immunizations were subsequently performed at 14-day intervals using FIA for emulsification, with a consistent dose of 0.5 mg per chicken for each inoculation. Before each immunization, peripheral blood was collected from the wing vein of chickens, and serum was isolated for detection of the specific antibody titer against VP1-CDEF' protein via ELISA. HRP-conjugated goat anti-chicken IgG H&L antibody (diluted at 1:5,000) was used as the secondary antibody, and serum collected from a third SPF chicken injected with PBS served as the negative control. The OD_450_ of each well was measured using a microplate reader. When the S/N ratio (OD_450_ of immune chicken serum/OD_450_ of negative control serum) reached ≥2.1 at a serum dilution of 1:64,000, peripheral blood was collected via the wing vein for isolation of peripheral blood mononuclear cells (PBMCs). PBMCs were immediately isolated using a chicken peripheral blood lymphocyte isolation kit (Solarbio, Beijing, China) per the manufacturer’s instructions. Isolated PBMCs were pooled and used to amplify the VH and VL genes.

### Amplification of immunoglobulin variable region genes and scFv assembly

Total RNA was extracted from the harvested PBMCs using the TRIzol reagent according to the manufacturer’s protocol. After verifying the purity and integrity of the extracted RNA, first-strand cDNA was synthesized using HiScript II Q RT SuperMix reagent (Vazyme, Nanjing, China). The VH and VL genes were amplified separately with the primers listed in Table 1, using the following thermal cycling conditions: initial denaturation at 95 °C for 3 min; 22 cycles of 95 °C for 25 s, 62 (or 65) °C for 25 s, and 72 °C for 30 s; and a final extension at 72 °C for 5 min. The purified VH and VL genes were mixed at an equimolar ratio and assembled into full-length scFv coding sequences by overlap extension PCR. This assembly was amplified using primers VH-F and VL-R (Table 1) with the following conditions: initial denaturation at 95 °C for 3 min; 18 cycles of 95 °C for 25 s, 60 (or 63)°C for 25 s, and 72 °C for 50 s; and a final extension at 72 °C for 5 min.

### Construction and diversity characterization of the scFv phage display library

The purified scFv gene fragments were ligated into the pComb3XSS phage vector, and the ligation mixture was electroporated into *E. coli* SS320 competent cells. The electroporated bacterial suspension was transferred to 30 mL of antibiotic-free 2YT medium and cultured at 37 °C for 1 h. M13KO7 helper phage was then added at a multiplicity of infection (MOI) of 10:1 (phage:bacteria), and the culture was incubated for an additional 1 h at 37 °C with gentle shaking to allow efficient infection. A 100 μL aliquot of the bacterial suspension was serially diluted 10-fold, spread onto LB agar plates containing ampicillin and kanamycin, and incubated overnight at 37 °C. Twenty single colonies were randomly selected, and colony PCR was performed using the pComb3XSS vector-specific primers (comb-F: 5′-CTTCCGGCTCGTATGTTGTGTGGAA-3′, comb-R: 5′-AGCCACCACCGGAAC CGCCTCCCTCA-3′) to confirm the presence of scFv inserts. Positive PCR products were sequenced to evaluate the sequence diversity of the scFv repertoire, and the capacity of the primary scFv library was calculated from the resulting colony count. The remaining bacterial culture was transferred into 300 mL of 2YT medium and incubated at 37 °C with shaking for 16–18 h. After centrifugation, the supernatant was collected, and a one-quarter volume of PEG 8000/NaCl solution was added, followed by thorough mixing by repeated inversion. The mixture was incubated on ice for 1 h and then centrifuged. The supernatant was discarded, and the phage pellet was resuspended in PBT buffer to obtain the primary phage-displayed scFv library.

### Biopanning of the scFv phage display library

ELISA plates were coated with 1 μg per well of VP1-CDEF' protein overnight at 4 °C; wells coated with PBS were set as blank controls. The next day, the plates were blocked with 5% skim milk for 1 h, after which 100 μL of the primary phage-displayed scFv library was added to each well. The plates were incubated at room temperature with shaking at 150 rpm for 2 h to allow antigen–antibody binding and then washed 10 times with PBS to remove unbound phages. Subsequently, 100 μL of 0.1 M HCl elution buffer was added to each well, and the plates were incubated with shaking at 150 rpm for 5 min to elute the specifically bound phages. The eluate was immediately neutralized by adding 40 μL of 1 M Tris–HCl (pH 9.1). The neutralized mixture was then added to an NEB 5α F′ bacterial culture at an OD_600_ of 0.2–0.4 and incubated for 1 h to allow infection. A 10 μL aliquot of the infected culture was serially diluted 10-fold and spotted onto LB agar plates containing ampicillin to determine the output titer. M13KO7 helper phage was added to the remaining infected culture, followed by further incubation for 1 h. The culture was then transferred into 35 mL of 2YT medium supplemented with antibiotics and incubated overnight. After phage precipitation with PEG 8000/NaCl solution, the first round of enriched phage-displayed scFv library was obtained. Two to three additional rounds of biopanning were performed following the same procedure to progressively enrich scFv-displaying phages specific for the VP1-CDEF' protein.

### Screening of phage-displayed scFv clones specific for VP1-CDEF' protein

The phage pool obtained after the third round of biopanning was serially diluted 10-fold and spread onto LB agar plates containing ampicillin and kanamycin. After overnight incubation at 37 °C, a total of 184 individual colonies were randomly picked and inoculated into 2YT medium supplemented with ampicillin and kanamycin in 96-deep-well plates. The plates were incubated at 37 °C with shaking until the cultures reached early log phase. M13KO7 helper phage was then added at an MOI of 10, and the mixture was incubated at 37 °C for 1 h with gentle shaking. Subsequently, the cultures were incubated overnight at 37 °C with shaking at 200 rpm and then centrifuged at 4,000 × g for 20 min at 4 °C. The resulting supernatants, containing phage-displayed scFv, were collected and used directly for ELISA screening.

ELISA plates were coated overnight at 4 °C with 100 ng per well of recombinant VP1-CDEF' protein. After blocking with 5% skim milk at 37 °C for 1 h, the plates were washed three times with PBST and then incubated with 100 μL of phage supernatant per well at 37 °C for 1 h. Following three additional washes with PBST, a 1:8,000 dilution of HRP-conjugated mouse anti-M13 antibody was added and incubated at 37 °C for 30 min. The plates were washed again, and TMB substrate was added. After color development, the reaction was stopped, and the absorbance at 450 nm (OD_450_) was measured. Clones with a sample-to-negative-control OD_450_ ratio (P/N) greater than 5 were identified as positive. The scFv-encoding genes from these positive clones were PCR-amplified using the comb-F and comb-R primer pair. The amplicons were subjected to Sanger sequencing, and the deduced amino acid sequences were aligned and analyzed.

### Eukaryotic expression of recombinant scFv-Fc protein and Western blot analysis

scFv clones with distinct amino acid sequences were selected, and their scFv genes were amplified using the primer pair scFv-F/scFv-R (Table 1). The amplified fragments were purified by agarose gel electrophoresis and cloned into the eukaryotic expression vector pFUSE-hIgG-Fc between the EcoRI and BsiWI restriction sites. The ligation mixture was introduced into *E. coli* DH5α competent cells by heat shock, and the transformed bacteria were spread onto LB agar plates containing the appropriate antibiotic and incubated overnight at 37 °C. Individual colonies were picked, and plasmids were extracted and verified by sequencing. Recombinant plasmids carrying the correct insert were used to transiently transfect Expi293F suspension cells. After 6 days of culture, the cell culture supernatants were harvested, and the scFv-Fc fusion protein was purified using a Protein A affinity chromatography column. The purified protein was analyzed by SDS-PAGE to confirm its molecular weight and purity. To evaluate the binding specificity of the recombinant scFv-Fc, VP1-CDEF' protein and FPV VP2 protein (serving as the target and a heterologous control, respectively) were resolved separately by SDS-PAGE and transferred onto PVDF membranes. Western blot was performed using the purified scFv-Fc as the primary antibody, followed by incubation with an HRP-conjugated mouse anti-human IgG-Fc antibody (diluted at 1:5,000) as the secondary antibody.

### Detection of the binding ability of scFv-Fc to FCV by indirect immunofluorescence assay (IFA)

F81 cells were seeded in a 96-well plate at a density of approximately 1 × 10^4^ cells/well. After 4–6 h of culture, the supernatant was removed, and the cells were infected with FCV at an MOI of 2.5 for 3 h. The viral inoculum was then discarded, and the cells were washed once with PBS. For fixation, 100 μL of 80% acetone (pre-cooled to −20 °C) was added to each well and incubated at −20 °C for 25 min, followed by three washes with PBS. Subsequently, 100 μL of recombinant scFv-Fc protein (50 μg/mL) was added per well and incubated at 37 °C for 1 h, followed by three washes with PBST. Cy3-conjugated goat anti-human IgG-Fc antibody (diluted at 1:500) was then applied and incubated at 37 °C for 1 h, followed by three additional washes with PBST. Nuclei were counterstained with Hoechst 33342 (5 μg/mL) for 10 min at room temperature in the dark, and then washed three times with PBS. Fluorescence images were acquired using a fluorescence microscope.

### Virion capture ELISA

FCV LZ2023 strain was propagated and purification as previously described (Conley et al., 2019). The virus concentration was determined using a BCA assay. The integrity of the purified virions was confirmed by negative-staining transmission electron microscopy (TEM). For virion capture ELISA, 96-well ELISA plates were coated with 100 μL of purified FCV virions (10 μg/mL in PBS) overnight at 4 °C. Plates coated with PBS alone or with purified FPV virions (prepared similarly) served as blank and negative-control antigens, respectively. After blocking with 5% skim milk in PBST for 1 h at 37 °C, the plates were washed three times with PBST and incubated with 100 μL of each purified scFv-Fc protein at a concentration of 10 μg/mL (in blocking buffer) for 1 h at 37 °C. Following five washes with PBST, HRP-conjugated goat anti-human IgG-Fc antibody (1:5,000 dilution) was added and incubated for 1 h at 37 °C. After extensive washing, TMB substrate was added, the reaction was stopped with 2 M H_2_SO_4_, and the absorbance was read at 450 nm. Each sample was tested in triplicate, and the experiment was repeated independently three times.

### Determination of the binding affinity between scFv-Fc and VP1-CDEF' protein by biolayer interferometry (BLI)

The binding affinity between scFv-Fc and VP1-CDEF' protein was measured using the Octet® R2 system (Sartorius, Göttingen, Germany). All assays were performed at 25 °C with continuous shaking at 1000 rpm. Protein A biosensors were pre-hydrated in kinetics buffer (PBS containing 0.02% Tween-20 and 0.1% BSA) for 10 min before use. In a 96-well black microplate, 220 μL of kinetics buffer was added to the baseline, association, and dissociation wells. Recombinant scFv-Fc protein diluted to 5 μg/mL in kinetics buffer was added to the loading wells for ligand immobilization onto the biosensors. A series of 2-fold serial dilutions of VP1-CDEF' protein in kinetics buffer was prepared and added to the analyte wells. The assay protocol included a 60 s baseline step, a 120 s loading step, a 60 s baseline step, a 180 s association step, and a 300 s dissociation step. After the run, the raw data were exported and processed using Octet Data Analysis Software 12.0. The equilibrium dissociation constant (KD) was calculated by fitting the data to a 1:1 Langmuir binding model.

### Statistical analysis

All experiments were performed at least in triplicate, and data are presented as mean ± SD unless otherwise noted. Comparisons between groups were performed using Student’s *t*-test (two-tailed) or one-way ANOVA, as appropriate. A *p* < 0.05 was considered statistically significant.

## Results

### Prokaryotic expression and affinity purification of VP1-CDEF' protein

The constructed pET-28a-VP1-CDEF' recombinant plasmid was verified by PCR. Agarose gel electrophoresis (Figure 1A) showed that the amplified target band was approximately 430 bp, consistent with the expected size of the VP1-CDEF' gene fragment. Sequencing results further confirmed that the recombinant plasmid was correctly constructed. The pET-28a-VP1-CDEF' plasmid was transformed into *E. coli* Rosetta (DE3) competent cells for induced expression. Following sonication, the cell lysate supernatant was collected, and the target protein was purified using Ni-NTA affinity chromatography. SDS-PAGE analysis under reducing conditions followed by Coomassie Brilliant Blue R-250 staining (Figure 1B) revealed a distinct ~18 kDa band in both the IPTG-induced bacterial lysate and the Ni-NTA-purified eluate fractions, consistent with the theoretical molecular weight of VP1-CDEF'. Western blot analysis (Figure 1C) demonstrated that the purified recombinant VP1-CDEF' protein exhibited a distinct specific immunoreactive band at the expected molecular weight of ~18 kDa, confirming the successful expression and antigenicity of the recombinant VP1-CDEF' protein.

**Figure 1:**
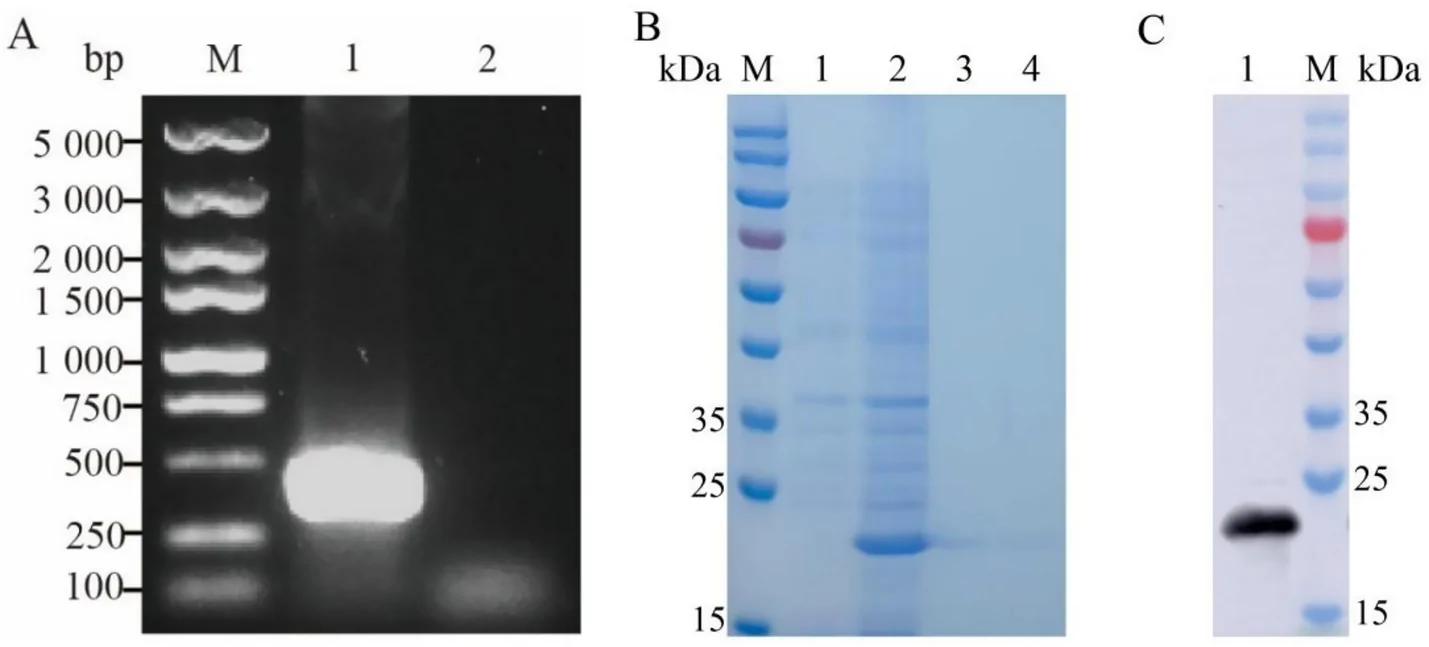
Construction, expression, purification, and identification of recombinant VP1-CDEF' protein. **(A)** Agarose gel electrophoresis analysis of PCR amplification products of the recombinant pET-28a-VP1-CDEF' plasmid. M: DL5000 DNA marker; 1: ~430 bp PCR product of the VP1-CDEF' gene; 2: Negative control. **(B)** SDS-PAGE analysis of VP1-CDEF' protein expression and purification. M: Protein molecular weight marker (180 kDa); 1: Uninduced bacterial lysate; 2: IPTG-induced bacterial lysate; 3–4: Ni-NTA-purified eluate fractions of VP1-CDEF' protein. **(C)** Western blot analysis of recombinant VP1-CDEF' protein antigenicity. M: Protein molecular weight marker; 1: Purified recombinant VP1-CDEF' protein.

### Kinetics of VP1-CDEF'-specific antibody responses in immunized chickens

To generate high-titer polyclonal antibodies against VP1-CDEF' for subsequent phage display library construction, two SPF chickens were immunized with purified recombinant VP1-CDEF' protein according to the immunization protocol described above. Serum samples were collected before each immunization, and the specific antibody titers against VP1-CDEF' were determined by indirect ELISA. As shown in Figure 2, the titration curves demonstrated that specific antibody levels increased progressively with successive immunizations, and peaked after the fifth immunization, with robust OD_450_ signals retained even at high serum dilutions. The two immunized chickens exhibited highly consistent immune responses with negligible individual variation, whereas serum from the PBS-injected negative control chicken showed persistently low background signals across all dilution gradients, confirming the specificity of the ELISA assay. Importantly, after the fifth immunization, the S/N ratios of both chicken A and chicken B exceeded 2.1 at a serum dilution of 1:64,000, meeting the predetermined threshold for antibody library construction.

**Figure 2:**
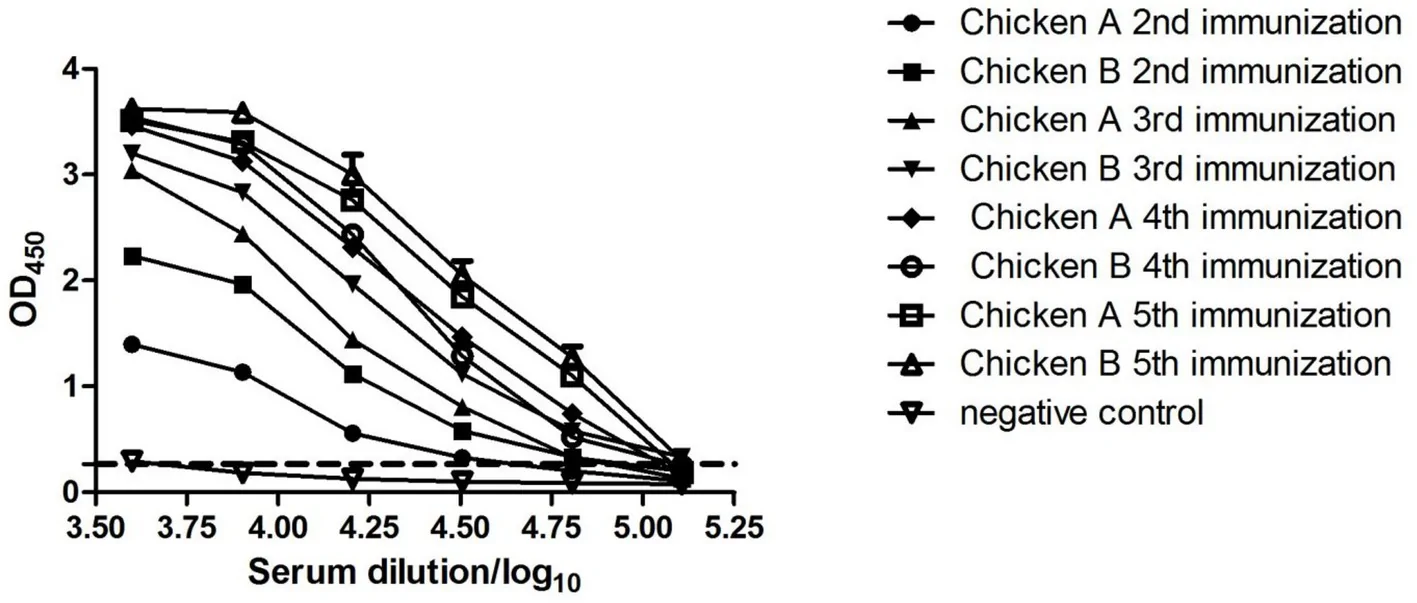
Kinetics of VP1-CDEF'-specific antibody responses in immunized chickens. Two SPF chickens received five immunizations with recombinant VP1-CDEF' protein in total. Serum samples were collected before each booster immunization and serially diluted for titer measurement. Chicken A and Chicken B: SPF chickens immunized with recombinant VP1-CDEF' protein; Negative control: SPF chicken injected with PBS. OD_450_ values represent the mean ± SD of triplicate measurements (*n* = 3). The *x*-axis represents serum dilution on a log_10_ scale, and the *y*-axis represents the optical density value at 450 nm (OD_450_). HRP-conjugated goat anti-chicken IgG H&L antibody at a dilution of 1:5,000 was used as the secondary antibody for detection. The dashed line denotes the cut-off value corresponding to an S/N ratio of 2.1, which is defined as the OD_450_ of immune serum divided by that of the negative control serum.

### Amplification of VH, VL and scFv genes

Peripheral blood was collected from both immunized chickens after the fifth immunization, and PBMCs were isolated and pooled for subsequent amplification of immunoglobulin variable region genes. Briefly, total RNA was extracted from the pooled PBMCs and used as the template for cDNA synthesis via reverse transcription. The VH and VL genes were amplified separately by PCR from the synthesized cDNA. Agarose gel electrophoresis analysis (Figures 3A,B) revealed distinct bands of the amplified products at ~420 bp and ~360 bp, respectively, consistent with the expected sizes of the VH and VL gene fragments. The purified VH and VL genes were then assembled into full-length scFv genes by overlap extension PCR. Agarose gel electrophoresis analysis revealed distinct specific bands of the assembled products at ~780 bp, confirming the successful construction of the scFv gene fragments (Figure 3C).

**Figure 3:**
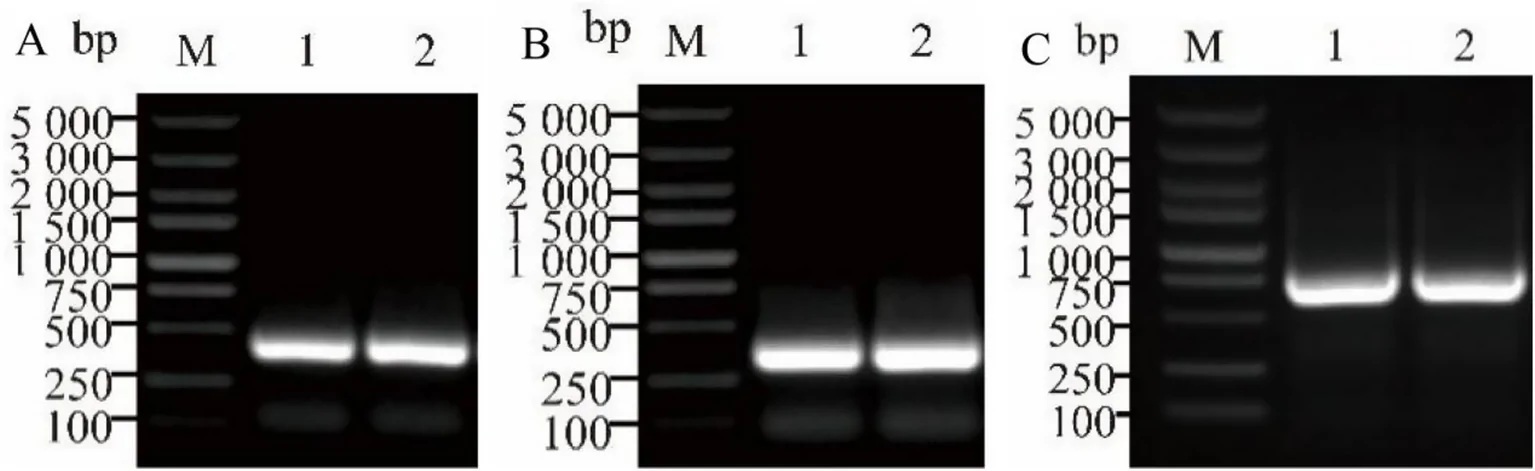
Amplification of chicken immunoglobulin variable region genes and assembly of scFv genes. **(A)** Agarose gel electrophoresis of VH gene amplification products. Lanes 1–2: ~420 bp VH gene fragments amplified at annealing temperatures of 62 °C and 65 °C, respectively. **(B)** VL gene amplification products. Lanes 1–2: ~360 bp VL gene fragments amplified at annealing temperatures of 62 °C and 65 °C, respectively. **(C)** Overlap extension PCR products for scFv assembly. Lanes 1–2: ~780 bp full-length scFv gene fragments assembled at annealing temperatures of 60 °C and 63 °C, respectively.

### Construction and quality assessment of the phage-displayed scFv antibody library

The purified scFv gene fragments were cloned into the pComb3XSS phagemid vector to construct the primary scFv phage-displayed library. Twenty single colonies were randomly selected from the library and analyzed by colony PCR. The results showed distinct bands of ~780 bp, consistent with the expected size of the scFv gene fragments. Of the 20 clones analyzed, 16 contained correct inserts, corresponding to a ligation efficiency of 80% (Figure 4A). The total size of the primary scFv phage-displayed library was 5.25 × 10^7^ independent transformants. Positive clones with correct inserts were subjected to Sanger sequencing. The deduced amino acid sequences were aligned using MEGA11 software, and a phylogenetic tree was constructed using the neighbor-joining method. Sequence alignment and phylogenetic analysis revealed high diversity in the immunoglobulin variable regions, confirming that the constructed antibody library exhibited good diversity (Figure 4B).

**Figure 4:**
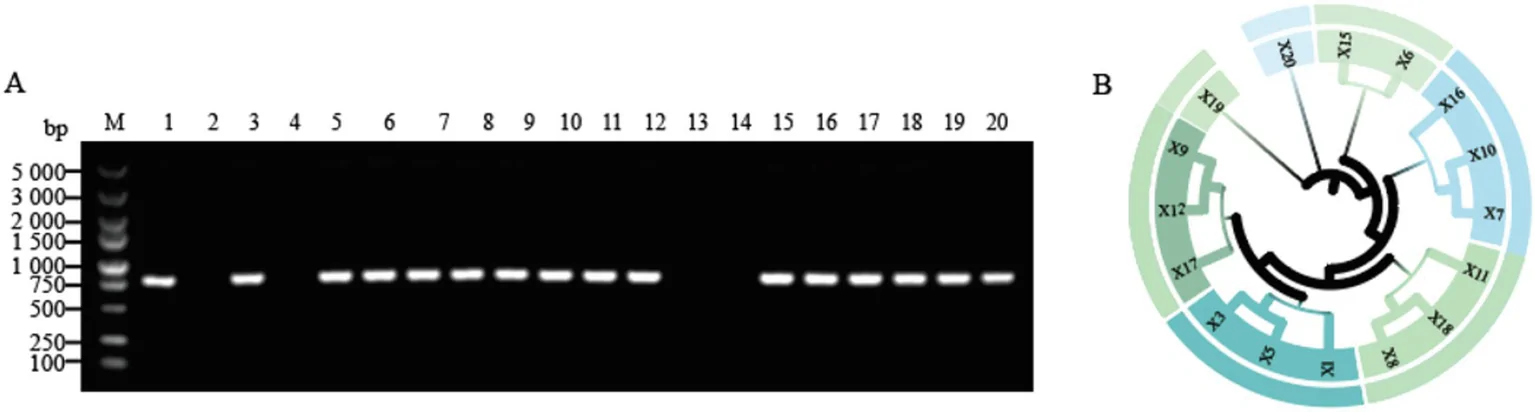
Quality characterization of the primary phage-displayed scFv antibody library. **(A)** Colony PCR analysis of scFv insert efficiency. Lanes 1–20: PCR products from randomly selected individual clones. Sixteen out of 20 clones showed the expected ~780 bp band, corresponding to an insert efficiency of 80%. **(B)** Phylogenetic tree of scFv variable region amino acid sequences. The tree was constructed using the neighbor-joining method in MEGA11 software based on the deduced amino acid sequences of 16 positive clones. The distinct branching pattern indicates high sequence diversity of the library.

### Panning of the phage-displayed scFv library

Using VP1-CDEF' protein as the target antigen, three rounds of biopanning were performed on the primary phage-displayed scFv library using a 96-well microplate-based method. Input and output phage titers were determined after each round to calculate the recovery rate and enrichment fold. As shown in Table 2, the phage recovery rate after elution increased progressively from 3.6 × 10^−6^ to 6.4 × 10^−3^, and the enrichment fold rose from 1.00 to 1777.78. After the third round, VP1-CDEF'-specific phages were markedly enriched compared with the antigen-free control, whereas non-specific phages were effectively depleted. These results demonstrate the successful and specific enrichment of VP1-CDEF'-binding phages from the phage-displayed scFv library.

**Table 2:** Enrichment of VP1-CDEF'-specific phages during three rounds of biopanning of the phage-displayed scFv library.

Item	First round	Second round	Third round
Input phage colony	2 × 10^7^	2 × 10^6^	2 × 10^6^
Output phage colony	72	2.74 × 10^3^	1.28 × 10^4^
Recovery rate (output/input)	3.6 × 10^−6^	1.37 × 10^−3^	6.4 × 10^−3^
Enrichment fold (current round/round 1)	1.00	380.6	1777.78

### Screening of VP1-CDEF'-specific scFv clones by phage ELISA

The phage pool obtained after the third round of biopanning was serially diluted and plated onto LB agar plates, followed by overnight incubation at 37 °C. A total of 184 single colonies were randomly selected for phage ELISA screening, with a P/N ratio > 5 used as the cutoff for positive clones. Based on the screening results (Figures 5A,B), 25 positive scFv clones that specifically bound to the VP1-CDEF' protein were identified. To further characterize these positive clones, colony PCR was performed using the pComb3XSS vector-specific primers comb-F and comb-R. The PCR products were purified using a gel extraction kit and subjected to Sanger sequencing. The obtained nucleotide sequences were translated into amino acid sequences and aligned using MEGA11 software. Sequence analysis revealed five distinct scFv antibodies with unique amino acid sequences, which were designated as 1A2, 2B2, 2C1, 2E1, and 2G5 (Figure 5C).

**Figure 5:**
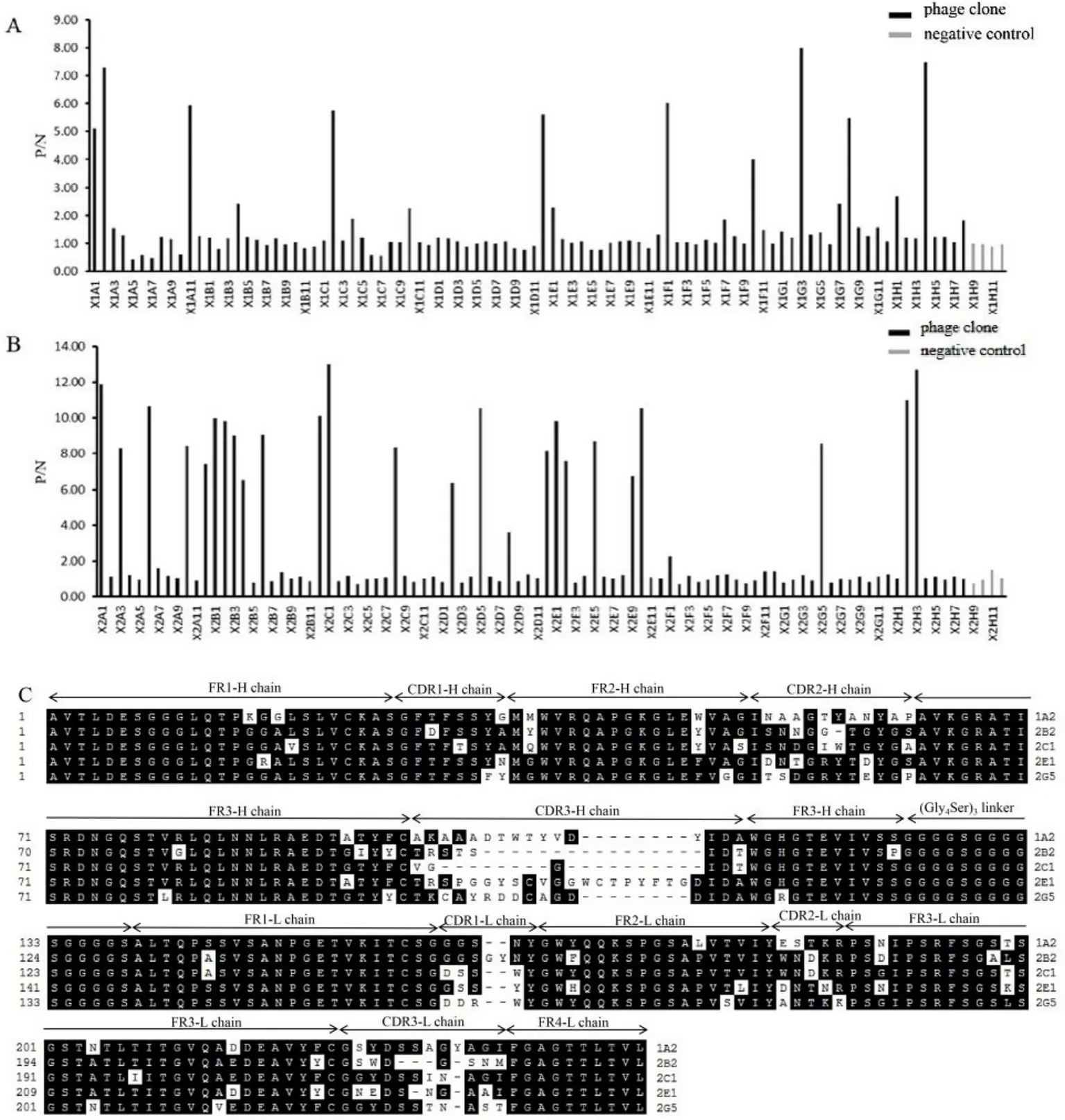
Identification and sequence analysis of VP1-CDEF'-specific scFv clones. **(A,B)** Phage ELISA screening of 184 randomly selected clones from the third round of biopanning. Clones with a P/N ratio > 5 were considered positive. A total of 25 positive clones were obtained. **(C)** Amino acid sequence alignment of the five unique scFv clones (1A2, 2B2, 2C1, 2E1 and 2G5) using MEGA11 software. The CDR and FR regions were identified according to IMGT rules for chicken Ig-VH and Ig-VL domains. The CDRs exhibit notable amino acid sequence diversity.

### Expression, purification and Western blot verification of recombinant scFv-Fc proteins

The scFv genes from the five positive clones 1A2, 2B2, 2C1, 2E1 and 2G5 were amplified by PCR. Agarose gel electrophoresis analysis revealed distinct bands of ~750 bp, confirming successful amplification of the target scFv genes. The gel-purified target genes were digested with the corresponding restriction enzymes and ligated into the eukaryotic expression vector pFUSE-hIgG-Fc to construct recombinant expression plasmids. The recombinant plasmids were transiently transfected into Expi293F suspension cells for secretory expression of scFv-Fc fusion proteins. The target proteins were then purified from the cell culture supernatants using Protein A affinity chromatography. SDS-PAGE analysis under reducing conditions showed that the purified proteins appeared as bands with a molecular weight of approximately 55 kDa, consistent with the theoretical molecular weight of scFv-Fc fusion proteins, confirming successful expression and purification of all five recombinant proteins (Figure 6A).

**Figure 6:**
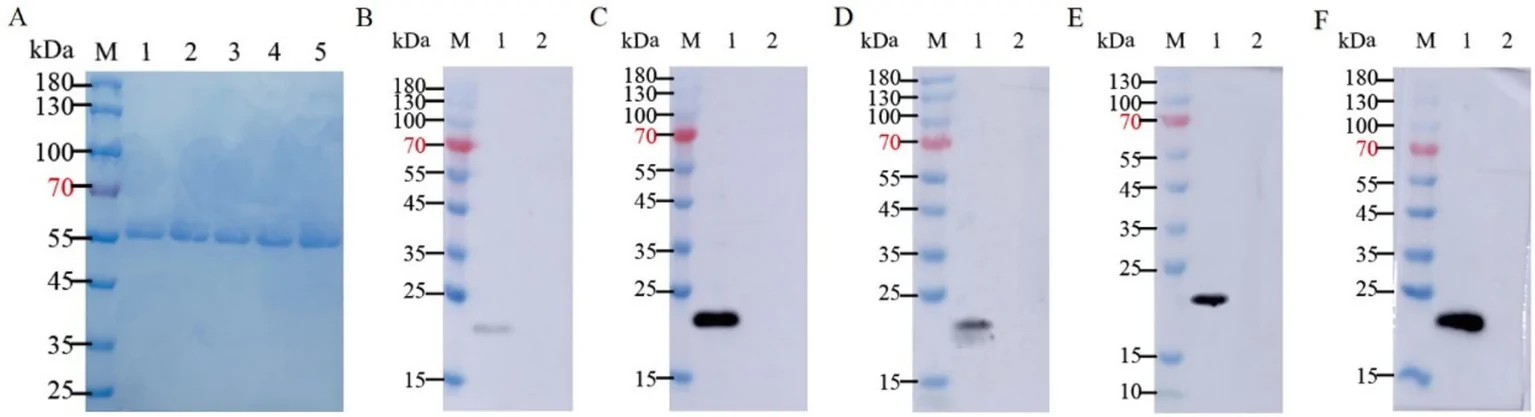
Expression, purification and antigen-binding specificity analysis of recombinant scFv-Fc fusion proteins. **(A)** 10% SDS-PAGE analysis under reducing conditions followed by Coomassie Brilliant Blue R-250 staining of purified scFv-Fc proteins. Lanes 1–5: Purified 1A2, 2B2, 2C1, 2E1 and 2G5 scFv-Fc proteins, respectively. All five proteins showed a single major band at ~55 kDa with high purity. **(B–F)** Western blot analysis of scFv-Fc binding specificity. Purified VP1-CDEF' and FPV VP2 proteins were separated by SDS-PAGE and transferred to PVDF membranes. The membranes were incubated with each scFv-Fc protein as the primary antibody: **(B)** 1A2, **(C)** 2B2, **(D)** 2C1, **(E)** 2E1, **(F)** 2G5. All five scFv-Fc proteins specifically recognized VP1-CDEF' protein with no cross-reactivity to the FPV VP2 negative control.

To verify the specific binding activity of the recombinant scFv-Fc proteins, Western blot analysis was performed. Purified VP1-CDEF' protein and FPV VP2 protein (negative control) were separated by SDS-PAGE and transferred to PVDF membranes. The membranes were incubated with each of the five purified scFv-Fc proteins as primary antibodies, followed by HRP-conjugated goat anti-human IgG-Fc secondary antibody. The results demonstrated that all five scFv-Fc proteins specifically bound to VP1-CDEF' protein and showed no cross-reactivity with FPV VP2 protein (Figures 6B–F).

### IFA detection of FCV-infected cells via recombinant scFv-Fc proteins

F81 cells were infected with the FCV LZ2023 strain at an MOI of 2.5. After 3 h of infection at 37 °C, the cells were fixed with ice-cold 80% acetone for 25 min at −20 °C. IFA was performed using the five purified scFv-Fc fusion proteins as primary antibodies and Cy3-conjugated goat anti-human IgG-Fc antibody as the secondary antibody. Fluorescence microscopy revealed distinct specific red fluorescent signals in the wells treated with 2B2-Fc, 2E1-Fc, and 2G5-Fc, indicating that these three scFv-Fc proteins could specifically recognize and bind to FCV-infected F81 cells. In contrast, no specific red fluorescent signals were detected in the 1A2-Fc and 2C1-Fc wells, suggesting that these two proteins exhibited no detectable binding to FCV. The negative control well, in which the primary antibody was replaced with PBS, showed no red fluorescent signal (Figure 7).

**Figure 7:**
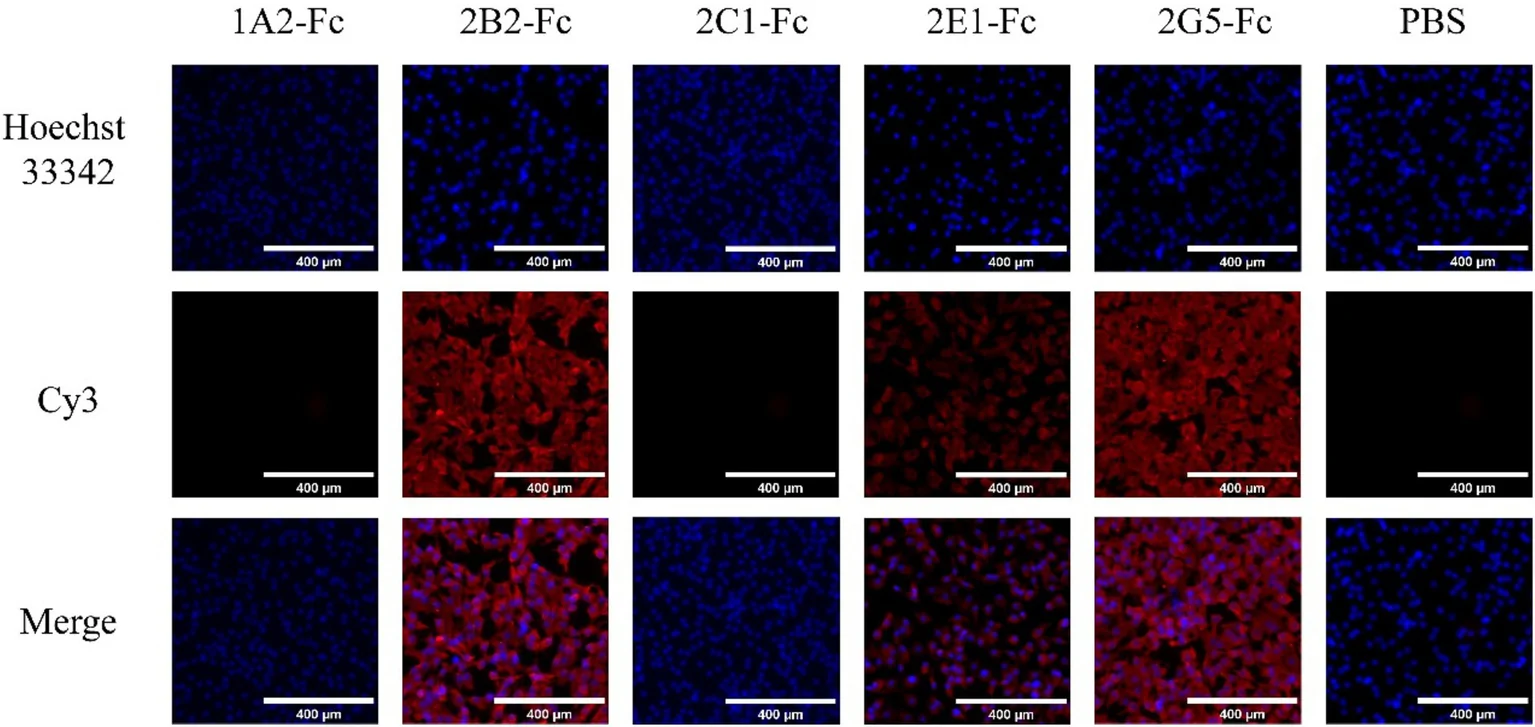
IFA detection of scFv-Fc fusion protein binding to FCV-infected F81 cells. F81 cells were infected with FCV LZ2023 strain at an MOI of 2.5 and fixed with ice-cold 80% acetone. The cells were incubated with each purified scFv-Fc protein (50 μg/mL) as the primary antibody, followed by Cy3-conjugated goat anti-human IgG-Fc secondary antibody (red). Nuclei were counterstained with Hoechst 33342 (blue). Columns from left to right: 1A2-Fc, 2B2-Fc, 2C1-Fc, 2E1-Fc, 2G5-Fc, and PBS negative control (primary antibody replaced with PBS). Scale bar = 400 μm for all panels. Distinct specific red fluorescent signals were observed in cells treated with 2B2-Fc, 2E1-Fc, and 2G5-Fc, while no specific signals were detected in the other groups.

### Virion capture ELISA confirmed binding to intact FCV virions

FCV LZ2023 virions were purified from infected F81 cell culture supernatants by centrifugation through a caesium chloride gradient (1.31–1.45 g/mL). The integrity of the purified virions was confirmed by negative-staining transmission electron microscopy (TEM), which showed intact, non-enveloped icosahedral particles of approximately 35–40 nm in diameter, consistent with the typical morphology of FCV (Figure 8A). The purified virion preparation was then used to coat ELISA plates for virion capture assays. As shown in Figure 8B, the virion capture ELISA results demonstrated that 2B2-Fc and 2E1-Fc showed strong binding to intact FCV virions (OD_450_ > 2 at 10 μg/mL), confirming that these two clones recognize surface-accessible epitopes on the native virus capsid. 2G5-Fc exhibited moderate but reproducible binding to intact virions (OD_450_ ≈ 1.0–1.5), consistent with its moderate affinity and its positive IFA staining. 1A2-Fc and 2C1-Fc showed no binding above background to intact virions (OD_450_ < 0.3), the two antibodies may recognize linear/denatured epitopes that are accessible on the recombinant VP1-CDEF' protein in Western blot but are buried or unavailable on the surface of the intact viral capsid. None of the scFv-Fc clones bound to the FPV virion negative control, confirming specificity.

**Figure 8:**
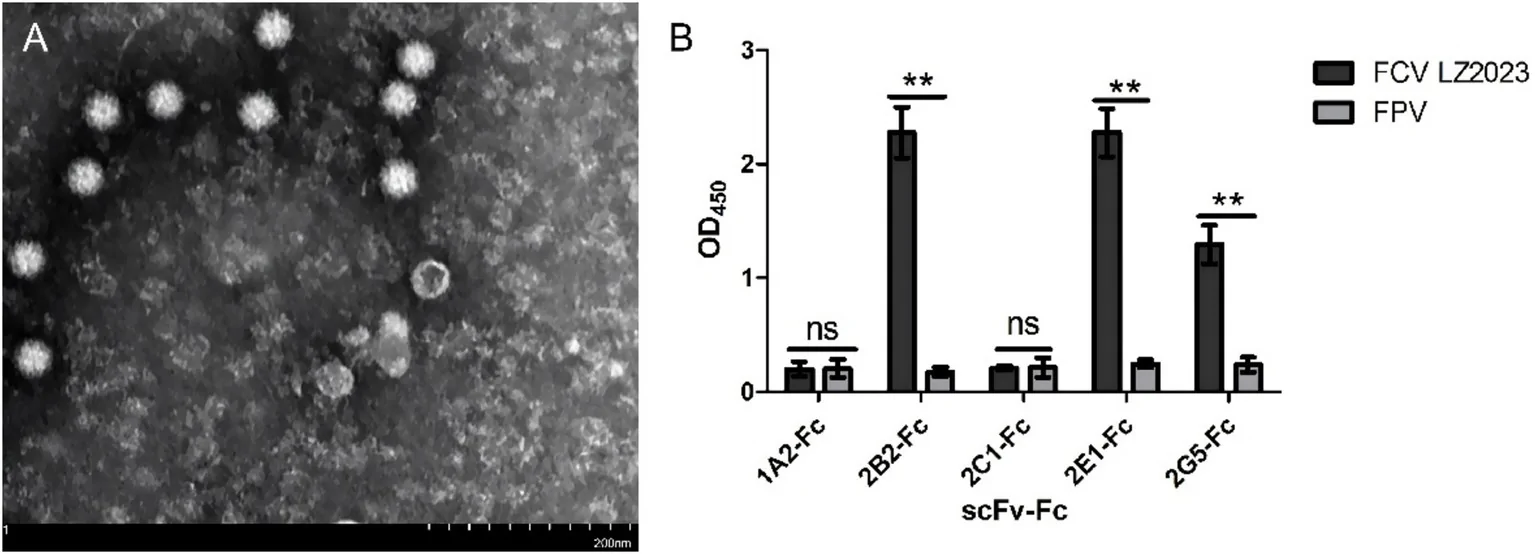
Binding reactivity of scFv-Fc fusion proteins to intact FCV virions analyzed by virion capture ELISA. **(A)** FCV LZ2023 strain was propagated in F81 cells and was purified by centrifugation through a caesium chloride gradient (1.31–1.45 g/mL). The integrity of the purified virions was confirmed by negative-staining transmission electron microscopy (TEM). **(B)** Purified intact FCV LZ2023 virions were coated on 96-well plates, with purified FPV virions included as the heterologous negative control. Each scFv-Fc protein was tested at a final concentration of 10 μg/mL. Binding signals were detected using HRP-conjugated goat anti-human IgG-Fc antibody and expressed as absorbance at 450 nm (OD_450_). Data are presented as mean ± SD from three independent experiments performed in triplicate. Statistical significance was determined by one-way repeated-measures ANOVA with Dunnett’s post-hoc test (*p < 0.05; **p < 0.01). None of the scFv-Fc clones showed detectable binding to FPV virions, confirming the target specificity of the antibodies.

### Affinity determination of scFv-Fc fusion proteins by BLI

The binding kinetics and equilibrium dissociation constants (KD) of the five scFv-Fc fusion proteins for VP1-CDEF' protein were determined using the Octet® R2 BLI system. As shown in Figure 9 and Table 3, both 2E1-Fc and 2B2-Fc exhibited high binding affinities, with KD values of 0.032 nM and 0.046 nM, respectively. 2G5-Fc displayed a moderate low-nanomolar affinity (KD = 65.44 nM), whereas 1A2-Fc and 2C1-Fc showed relatively weak binding (KD = 1,054 nM and 451 nM, respectively). These affinity results were consistent with the Western blot and IFA findings, further confirming the superior binding properties of 2B2-Fc, 2E1-Fc, and 2G5-Fc.

**Figure 9:**
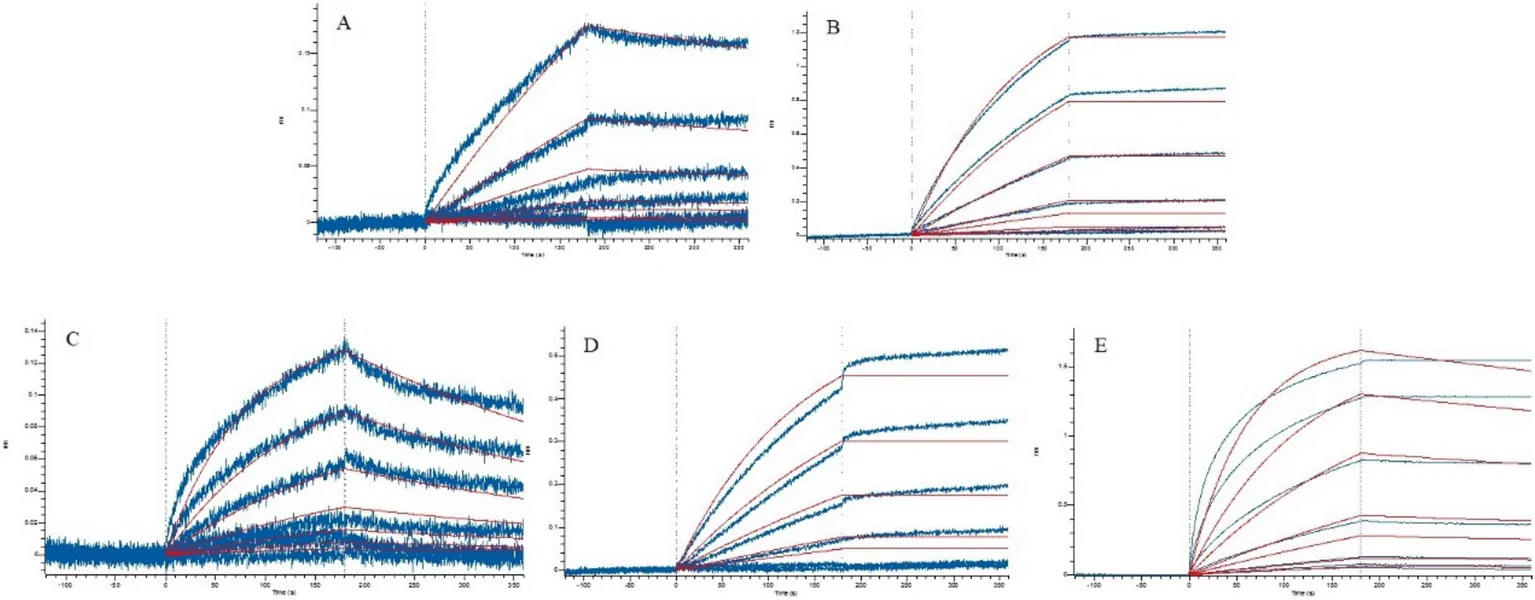
Binding kinetics of VP1-CDEF'-specific scFv-Fc fusion proteins determined by BLI. Purified scFv-Fc proteins were captured on Protein A biosensors, and serial dilutions of recombinant VP1-CDEF' protein were used as analytes. Sensorgrams show the association and dissociation phases of **(A)** 1A2-Fc, **(B)** 2B2-Fc, **(C)** 2C1-Fc, **(D)** 2E1-Fc, and **(E)** 2G5-Fc binding to VP1-CDEF' protein. Blue lines represent raw experimental data; red lines represent the global fitting of the data to a 1:1 Langmuir binding model.

**Table 3:** Binding affinities (KD) of VP1-CDEF'-specific scFv-Fc fusion proteins determined by BLI.

scFv-Fc clone	1A2-Fc	2B2-Fc	2C1-Fc	2E1-Fc	2G5-Fc
KD (nM)	1,054	0.046	451	0.032	65.44

## Discussion

FCV remains one of the most prevalent and clinically significant pathogens affecting felids worldwide. Despite widespread vaccination, the genetic and antigenic variability of FCV, particularly within the VP1 capsid protein, continues to compromise vaccine efficacy, underscoring the urgent need for rapid antibody-based diagnostic tools.(Wei et al., 2024). This study reports the generation of chicken-derived scFv antibodies targeting the FCV VP1-CDEF' region. The high-affinity clones 2B2 and 2E1 represent promising candidate reagents that warrant further evaluation for potential diagnostic applications.

The use of chickens as the antibody source offers distinct advantages over conventional mammalian systems. Due to the significant phylogenetic distance between avian and mammalian species, chickens mount robust immune responses against conserved mammalian antigens that might otherwise be tolerated in mammalian hosts (Capota et al., 2025). Furthermore, the chicken immunoglobulin gene organization, relying on gene conversion rather than complex V(D)J recombination, enables the amplification of full VH and VL repertoires using minimal primer sets, substantially simplifying library construction workflows (van Wyngaardt et al., 2004). In this study, we exploited these unique biological properties to construct a chicken-origin naive scFv library with a capacity of 5.25 × 10^7^ transformants and demonstrated good sequence diversity through phylogenetic analysis. The ligation efficiency (~80%) and the progressive enrichment of specific phages over three rounds of biopanning (enrichment fold reaching 1,777.78) underscore the technical feasibility of this approach.

Phage display technology has emerged as a powerful platform for rapid antibody discovery, particularly in veterinary diagnostics and therapeutics where traditional hybridoma methods are time-consuming and costly (Gong et al., 2024). Our biopanning strategy successfully enriched VP1-CDEF'-specific phage clones, yielding 25 positive clones from 184 screened colonies. Sequence analysis revealed five distinct scFv antibodies (1A2, 2B2, 2C1, 2E1, and 2G5), indicating that the library captured a broad repertoire of antigen-specific B-cell responses. Notably, the conversion of scFvs into scFv-Fc fusion proteins facilitated their expression in mammalian cells and enabled the use of standardized secondary detection reagents, a strategy previously validated in rabies virus and SARS-CoV-2 antibody development (Ray et al., 2001).

The binding characteristics of the five scFv-Fc clones exhibited marked heterogeneity. BLI analysis demonstrated that 2B2-Fc and 2E1-Fc possessed exceptionally high affinities for VP1-CDEF', with KD values of 0.046 nM and 0.032 nM, respectively. 2G5-Fc showed moderate affinity (KD = 65.44 nM), whereas 1A2-Fc and 2C1-Fc displayed relatively weak binding (KD > 450 nM). These differences likely reflect variations in CDR sequences and paratope architectures. Importantly, the high-affinity clones (2B2, 2E1, 2G5) not only bound recombinant antigen in Western blot but also efficiently recognized FCV virions in IFA, producing clear red fluorescent signals in infected F81 cells. In contrast, 1A2-Fc and 2C1-Fc failed to generate detectable red fluorescent signals despite weak Western blot reactivity, suggesting that affinity thresholds above a certain threshold are required for effective binding to epitopes on intact virions. It is important to note that the recombinant VP1-CDEF' protein used in this study was produced in *E. coli* and, as such, may not fully recapitulate the native conformation of the VP1 protein as it exists on the intact virus capsid. Western blot analysis primarily detects linear or denaturation-resistant epitopes, whereas IFA and virion capture ELISA detect epitopes that are accessible on the native viral particle. This distinction is clearly illustrated by clones 1A2 and 2C1, which recognized the denatured recombinant protein but failed to bind to intact virions or FCV-infected cells in IFA. These findings underscore the importance of using multiple orthogonal assays to characterize antibody binding specificity and highlight the need for native-antigen validation when developing diagnostic reagents. The discrepancy between the moderate affinity of 2G5-Fc and its robust signals in endpoint assays such as Western blot and IFA can be attributed to the fact that these assays are performed under saturating antibody concentrations and non-equilibrium conditions, which do not reflect the real-time dissociation kinetics measured by BLI. Additionally, epitope accessibility and potential avidity effects in solid-phase assays may contribute to the observed signal strength.

Three limitations of this study should be acknowledged. First, although we demonstrated specific binding of the developed scFv-Fc antibodies to the FCV genotype II LZ2023 strain, representing an initial proof-of-concept for the chicken-derived scFv platform, their cross-reactivity against other FCV genotypes has not been determined. Given the sequence variation in the E region among field isolates (Liang et al., 2022), future studies should evaluate the breadth of reactivity using a panel of genetically diverse FCV strains. Second, the neutralizing activity of these antibodies was not assessed in this work. While the VP1-CDEF' region harbors known neutralizing epitopes, not all antibodies targeting this region confer neutralizing capacity; some may bind non-neutralizing epitopes within the same domain (Cubillos-Zapata et al., 2020). Functional neutralization assays, such as plaque reduction or virus neutralization tests, are essential to establish the protective potential of candidate antibodies including 2B2 and 2E1. Third, although 2B2-Fc and 2E1-Fc exhibited high binding affinity and specificity for the VP1-CDEF' antigen and FCV-infected cells, the present study did not evaluate their performance in actual diagnostic formats. Lateral-flow immunochromatographic assays, detection-limit studies using purified virus or clinical samples, and cross-reactivity testing with other feline pathogens are essential prerequisites for validating the diagnostic potential of these antibodies. Therefore, while our findings provide a strong foundation for future development, the antibodies described herein should be considered as candidate reagents rather than validated diagnostic tools at this stage.

## Conclusion

In summary, we have successfully generated chicken-derived scFv antibodies against the VP1-CDEF' region of genotype II FCV LZ2023. Clones 2B2 and 2E1 exhibited high-affinity binding to the recombinant antigen and recognized viral antigens in FCV-infected cells. These antibodies are promising candidates for future diagnostic applications, but further validation in appropriate assay formats and with clinical specimens is required before they can be considered for practical use.

## Data Availability

The original contributions presented in the study are included in the article/supplementary material, further inquiries can be directed to the corresponding authors.
